# An inverse association of dietary choline with atherosclerotic cardiovascular disease among US adults: a cross-sectional NHANES analysis

**DOI:** 10.1186/s12889-024-18837-8

**Published:** 2024-05-31

**Authors:** Hui Lin, Zuoquan Zhong, Chuanjin Zhang, Xiaojun Jin, Xuchen Qi, Jiangfang Lian

**Affiliations:** 1 Department of Cardiology, The Affiliated Lihuili Hospital of Ningbo University Health Science Center, Ningbo, Zhejiang 315211 China; 2 Department of Respiratory Medicine, Shaoxing People’s Hospital, Shaoxing, China; 3 Department of Neurosurgery, Sir Run Run Shaw Hospital, Zhejiang University School of Medicine, Hangzhou, China; 4 Department of Neurosurgery, Shaoxing People’s Hospital, Shaoxing, China

**Keywords:** Atherosclerotic cardiovascular disease, Choline, Metabolic syndrome, NHANES

## Abstract

**Background:**

The role of diet choline in atherosclerotic cardiovascular disease (ASCVD) is uncertain. Findings from animal experiments are contradictory while there is a lack of clinical investigations. This study aimed to investigate the association between choline intake and ASCVD based on individuals from the National Health and Nutrition Examination Survey (NHANES) database.

**Methods:**

This cross-sectional study was conducted in 5525 individuals from the NHANES between 2011 and 2018. Participants were categorized into the ASCVD (*n* = 5015) and non-ASCVD (*n* = 510) groups. Univariable and multivariable-adjusted regression analyses were employed to investigate the relationship between diet choline and pertinent covariates. Logistic regression analysis and restricted cubic spline analysis were used to evaluate the association between choline intake and ASCVD.

**Results:**

ASCVD participants had higher choline intake compared to those without ASCVD. In the higher tertiles of choline intake, there was a greater proportion of males, married individuals, highly educated individuals, and those with increased physical activity, but a lower proportion of smokers and drinkers. In the higher tertiles of choline intake, a lower proportion of individuals had a history of congestive heart failure and stroke. After adjusting for age, gender, race, ethnicity, and physical activity, an inverse association between choline intake and heart disease, stroke, and ASCVD was found. A restricted cubic spline analysis showed a mirrored J-shaped relationship between choline and ASCVD, stroke and congestive heart failure in males. There was no association between dietary choline and metabolic syndrome.

**Conclusion:**

An inverse association was observed between choline intake and ASVCD among U.S. adults. Further large longitudinal studies are needed to test the causal relationship of choline and ASVCD.

**Supplementary Information:**

The online version contains supplementary material available at 10.1186/s12889-024-18837-8.

## Introduction

Atherosclerotic cardiovascular disease (ASCVD) is the leading cause of death in the United States. While traditional risk factors for ASCVD have been extensively researched [1], environmental risk factors (such as diet), which play an important role in the development ASCVD, are less well studied [2]. Choline is an essential nutrient for humans, but has limited endogenous synthesis and is concentrated in high-protein foods [3], including beef, fish, milk, eggs, cruciferous vegetables, and certain beans [4]. The current viewpoints on the role of choline on ASCVD are contradictory. Investigations demonstrated a central role for the choline to trimethylamine N-oxide (TMAO) pathway contributing to increased heart failure susceptibility [5, 6]. The evidence comes from experimental studies showing that a high-choline diet exacerbates cardiac dysfunction [7, 8] and that consumption of foods rich in choline are positively associated with stroke onset via production of TMAO in experimental and clinical stroke [9]. Conversely, choline was found to ameliorate cardiac hypertrophy by regulating metabolic remodeling [10, 11]. Addition of choline is able to increase neuroplasticity and recovery after stroke [12]. Currently, the exact role of diet choline in the development of ASCVD remains to be clarified.

Metabolic syndrome (MetS) is a multifactorial condition that increases the risk of ASCVD [13, 14]. It is characterized by central obesity, high fasting glucose, atherogenic dyslipidemia (high triglyceride, low high density lipoprotein cholesterol), and high blood pressure (BP) [15]. An estimated 20-30% of adults of the world have MetS. It is associated with increased risk of stroke, coronary heart disease, myocardial infarction and type 2 diabetes mellitus [16]. And recently, ABBASI el al. suggested higher dietary intakes of choline are associated with lower blood pressure levels among obese individuals [17]. However, other researchers found there was no association of choline intake with systolic or diastolic blood pressure [18, 19]. Due to the vital role of MetS in atherosclerosis progression, the real relationship between choline and MetS needs to be further investigated.

Therefore, the first objective of this study is to answer the clinical question: what is the exact relationship between choline intake and risks of ASCVD in adults. The second objective is to assess the contribution of choline to MetS and its components. The goal of the study is to provide a better understanding of the dietary risk factors for ASCVD.

## Methods

### Study design and population

**Fig. 1:**
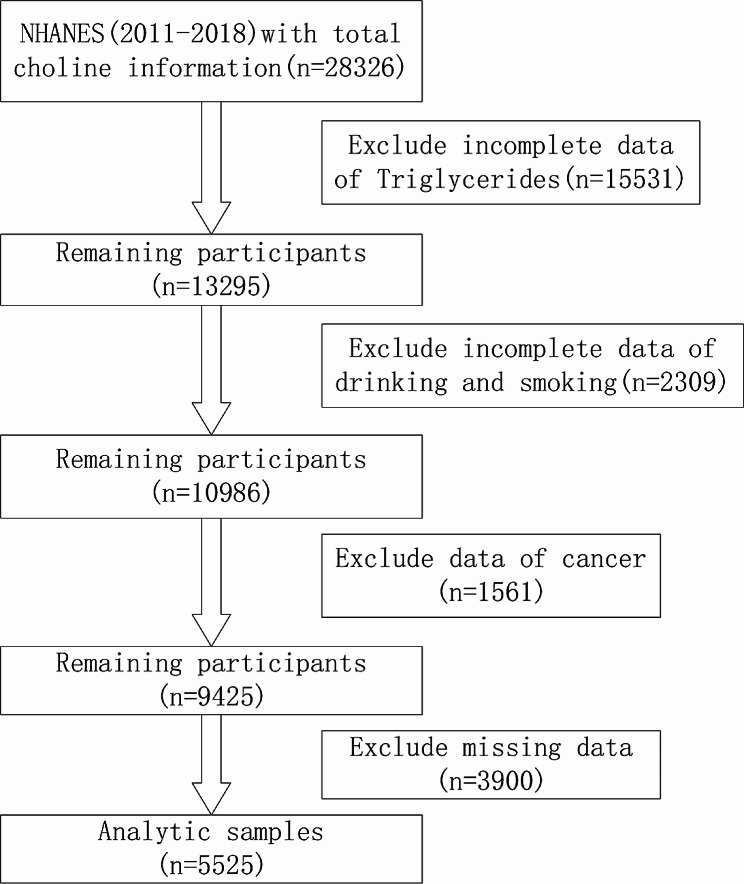
Flowchart of participant selection

The National Center for Health Statistics within the Centers for Disease Control and Prevention (CDC) conducts the National Health and Nutrition Examination Survey (NHANES), in order to evaluate the health and nutritional wellbeing of the American population through a series of cross-sectional surveys [20]. NHANES was approved by the National Center for Health Statistics Ethics Review Board and signed informed consent was obtained from all participants over 18 years old. For this analysis, the study population included 22,617 adults over 20 years old from 10 cycles of the NHANES from 2011 to 2018 (limited cycle due to the COVID-19 pandemic). The sample selection flowchart from NHANES is illustrated in Fig. 1. NHANES data can be viewed and accessed through the CDC–NCHS website (http://www.mayoclinicproceedings.org).

### Measurements of choline intake

The dietary choline intake of each participant was assessed by analyzing their consumption of all foods and beverages through two 24-hour dietary recall interviews, utilizing the Food and Nutrient Database for Dietary Studies provided by the United States Department of Agriculture. NHANES gathers two sets of 24-hour dietary recall data. The first interview is conducted in person at the Mobile Examination Center (MEC), while the second interview is conducted via telephone 3 to 10 days later [21]. We computed the mean dietary choline intake from the two 24-hour dietary surveys for additional analysis, as previously described [22]. The choline content of common foods are briefly listed in Supplementary Table 1, based on the United States Department of Agriculture (USDA) food content databases [23].

### Definition of ASCVD

ASCVD is the outcome of this study. ASCVD was diagnosed based on the presence of at least one of the following factors: congestive heart failure, coronary heart disease, angina/angina pectoris, heart attack, and stroke. All of above information is listed in the questionnaire at the CDC–NCHS website.

### Definition of metabolic syndrome (MetS)

The National Cholesterol Education Program Adult Treatment Panel III guidelines, revised in 2005, provided the following definition of MetS: presence of at least three of the following : (1) fasting plasma glucose ≥ 110 mg/dL; (2) systolic blood pressure (SBP) ≥ 130 mmHg or diastolic blood pressure (DBP) ≥ 85 mmHg; (3) serum triglycerides ≥ 150 mg/dL; (4) low serum high density lipoprotein cholesterol (HDL-C) < 40 mg/dL in men and < 50 mg/dL in women; (5) waist circumference ≥ 102 cm in men and ≥ 88 cm in women.

### Covariates

Covariates about individual characteristics included age, sex, race or ethnicity, education level, smoking status, and drinking status. Individuals who were taking medications to reduce their blood pressure, plasma glucose, or improve their lipid profile were categorized as having hypertension, diabetes, or dyslipidemia, respectively, regardless of their measured values for blood pressure, plasma glucose, or serum lipids. Physicians measured arterial blood pressure three times to determine the average value. Plasma glucose levels were evaluated in NHANES using a modified hexokinase enzymatic method. Body Mass Index was calculated as weight divided by height squared. Enzymatic methods were used to measure serum triglycerides, and low HDL-C levels were assessed by precipitating other lipoproteins with a mixture of polyanions and divalent cations. Total daily energy intake (kcal) was recorded. All detailed measurement procedures are available at www.cdc.gov/nchs/nhanes/publicly available. The detailed definition of disease (containing angina/angina pectoris, heart attack, coronary heart disease, hypertension and stroke) from NHANES database are shown in Supplementary Table 2.

### Statistical analysis

All analyses were performed with R (version 4.3.0) and accounted for the complex sampling design of the NHANES. To evaluate the demographic characteristics of participants based on their history of ASCVD, chi-square tests and t-tests were employed. The choline intake was divided into four quartiles and treated as categorical variables. We utilized multivariable logistic regression to determine the odds ratios (OR) and corresponding 95% confidence intervals (CI) for the relationships between choline intake and ASCVD or its components. Additionally, we employed multivariable linear regression to investigate the linear associations between choline intake and ASCVD. All analyses adjusted covariates for age, sex, race/ethnicity (Mexican American, other Hispanic, non-Hispanic White, non-Hispanic Black, or others), marital status (not living alone or living alone), physical activity (moderate or vigorous), drinking status (never or current drinker), and smoking status (never or current smoker).

To investigate potential sex differences, we conducted sex-stratified analyses in the logistic regression models and included the interaction term between sex and choline intake. To examine the shape of the dose-response relationships between choline intake and the risk of ASCVD, we conducted a restricted cubic spline analysis with 3-knots (25th, 50th, and 75th percentiles), using the median of choline levels as the reference point. We utilized the ANOVA function in the R rms package to estimate P_overall_ and P_nonlinear_, which indicates the statistical significance of the dose-response relationships. A value of less than 0.05 for both P_overall_ and P_nonlinear_ indicates a non-linear dose-response relationship. If only P_overall_ was less than 0.05, it indicates a linear dose-response relationship.

## Results

### Baseline characteristics of study participants

In this analysis, a total of 5525 participants were included, consisting of 510 ASCVD patients and 5015 individuals without ASCVD. The detailed demographic characteristics of the participants are presented in Table 1. The mean age of eligible participants was 48.0 ± 16.7 years. The proportion of men and women were roughly equal (51.5% vs. 48.5%). Non-Hispanic White accounted for the largest proportion (39.9%), followed by Non-Hispanic Black (22.6%), other races (14.1%), Mexican American (13.2%), and Other Hispanic (10.2%). Most participants were married (60.2%) and the educational level was above high school (59.1%). The participants had a relatively low physical activity level (22.1% of vigorous activity and 39.3% of moderate activity).

**Table 1:** Demographic characteristic of participants by presence of atherosclerotic cardiovascular diseases (ASCVD) in NHANES 2011–2018

	Total (*n* = 5525)	No ASCVD (*n* = 5015)	ASCVD (*n* = 510)	*P* value
Age [years, mean (SD)]	48.0 ± 16.8	46.5 ± 16.2	64.6 ± 12.1	< 0.001
Gender, n(%)				
Male	2845 (51.5%)	2547 (50.8%)	298 (58.4%)	< 0.001
Female	2680 (48.5%)	2468 (49.2%)	212 (41.6%)	
Race/ethnicity, n(%)				
Mexican American	727 (13.2%)	688 (13.7%)	39 (7.6%)	< 0.001
Other Hispanic	565 (10.2%)	520 (10.4%)	45 (8.8%)	
Non-Hispanic White	2204 (39.9%)	1945 (38.8%)	259 (50.8%)	
Non-Hispanic Black	1250 (22.6%)	1111 (22.2%)	139 (27.3%)	
Other	779 (14.1%)	751 (15.9%)	28 (5.5%)	
Marital status, n(%)				
Not living alone	3328 (60.2%)	3024 (60.3%)	304 (59.6%)	0.761
Living alone	2197 (39.8%)	1991 (39.7%)	206 (40.4%)	
Educational level, n(%)				
Below high school	347 (6.3%)	295 (5.9%)	52 (10.2%)	< 0.001
High school	1910 (34.6%)	1693 (33.8%)	217 (42.6%)	
Above high school	3268 (59.1%)	3027 (60.3%)	241 (47.2%)	
Smoking status, n(%)				
Never smoker	2914 (52.7%)	2741 (54.7%)	173 (33.9%)	< 0.001
Current smoker	2611 (47.3%)	2274 (45.3%)	337 (66.1%)	
Drinking status, n(%)				
Never drinker	1080 (19.6%)	884 (17.6%)	196 (38.4%)	< 0.001
Current drinker	4445 (80.4%)	4131 (82.4%)	314 (61.6%)	
Body Mass Index (kg/m^2^)	28.2 (24.5, 33.0)	28.0 (24.4, 32.8)	29.7 (26.0, 34.7)	< 0.001
Waist circumference (cm)	98.5 (88.2, 109.7)	97.5 (87.3, 108.7)	105.1 (96.5, 116.6)	< 0.001
Triglycerides (mg/dl)	97 (65, 144)	96 (65, 143)	107 (75, 151)	< 0.001
Fasting glucose (mg/dl)	101 (94, 111)	100 (94, 110)	108 (98, 130)	< 0.001
High density cholesterol (mg/dl)	51 (42, 62)	51 (43, 62)	48 (40, 60)	< 0.001
Systolic Blood pressure (mmHg)	120.7 (111.3, 132.7)	120 (110.7, 131.3)	129.2 (117, 142.7)	< 0.001
Diastolic Blood pressure (mmHg)	70.7 (64, 77.3)	71 (64, 78)	68 (60-73.3)	< 0.001
Total energy intake (kcals)	1861 (1357, 2465)	1874 (1373, 2498)	1696 (1228, 2260)	< 0.001
Choline intake (mg/day)	287.5 (188.9-416.8)	288.9 (190.6-417.8)	269.55 (172.25–408.40)	0.029
Vigorous activity, n(%)				
Yes	1224 (22.2%)	1140 (22.7%)	84 (16.5%)	0.001
No	4301 (77.8%)	3875 (77.3%)	426 (82.5%)	
Moderate activity, n(%)				
Yes	2169 (39.3%)	2000 (39.9%)	169 (33.1%)	0.003
No	3356 (60.7%)	3015 (60.1%)	341 (66.9%)	

### Relationship between choline and ASCVD

Significant differences were observed between the ASCVD and non-ASCVD groups in terms of age, gender, race/ethnicity, marital status, educational level, smoking status, drinking status, and intensity of physical activity. Regarding continuous variables, individuals with ASCVD had higher levels of triglycerides, fasting glucose, BP (both systolic and diastolic), and lower levels of HDL-C compared to those without ASCVD. In addition, we found the amounts of choline intake were also significantly higher in ASCVD participants than those without (289 vs. 270 mg/d, *P* = 0.029). To investigate the association between choline intake and ASCVD and its risk factors, we have presented the distribution of choline intake levels (represented by interquartile range) among ASCVD and non-ASCVD participants. As shown in Table 2, in higher tertiles of choline, there was a higher proportion of persons who were male, married, older, and highly educated, but a lower proportion were smokers and drinkers. In addition, there was a higher level of physical activity in higher tertiles of dietary choline (*P* < 0.01). In terms of ASCVD, there was a lower proportion of congestive heart failure (*P* = 0.02) and stroke (*P* = 0.003) in higher tertiles of choline intake. No significant difference were observed in history of coronary heart disease, angina/angina pectoris and heart attacks (Table 3).

**Table 2:** Characteristics of study population according to quartiles of choline intake

	Quartile1 (*n* = 1384)	Quartile2(*n* = 1380)	Quartile3(*n* = 1380)	Quartile4(*n* = 1381)	*P* for trend
Age [mean (SD)]	47.2 ± 17.4	48.4 ± 17.0	49.3 ± 16.6	47.3 ± 16.0	0.002
Gender, n(%)					
Male	495 (35.8%)	644 (46.7%)	760 (55.1%)	946 (68.5%)	< 0.001
Female	889 (64.2%)	736 (53.3%)	620 (44.9%)	435 (31.5%)	
Race/ethnicity, n(%)					
Mexican American	172 (12.4%)	151 (11.0%)	196 (14.2%)	208 (15.1%)	0.011
Other Hispanic	131 (9.5%)	171 (12.4%)	134 (9.7%)	129 (9.3%)	
Non-Hispanic White	563 (40.7%)	555 (40.2%)	550 (39.9%)	536 (38.8%)	
Non-Hispanic Black	338 (24.4%)	315 (22.8%)	292 (21.1%)	305 (22.1%)	
Other	180 (13.0%)	188 (13.6%)	208 (15.1%)	203 (14.7%)	
Marital status, n(%)					
Not living alone	741 (53.5%)	823 (59.6%)	881 (63.8%)	883 (63.9%)	< 0.001
Living alone	643 (46.5%)	557 (40.4%)	499 (36.2%)	498 (36.1%)	
Educational level, n(%)					
Below high school	104 (7.5%)	82 (5.9%)	83 (6.0%)	78 (5.7%)	< 0.001
High school	549 (39.7%)	443 (32.1%)	439 (31.8%)	489 (35.4%)	
Above high school	731 (52.8%)	855 (62.0%)	858 (62.2%)	814 (58.9%)	
Smoking status, n(%)					
Never smoker	729 (52.7%)	612 (44.3%)	748 (54.2%)	669 (48.4%)	< 0.001
Current smoker	655 (47.3%)	768 (55.7%)	632 (45.8%)	712 (51.6%)	
Drinking status, n(%)					
Never drinker	309 (52.7%)	301 (21.8%)	232 (16.8%)	238 (17.2%)	< 0.001
Current drinker	1075 (47.3%)	1079 (78.2%)	1148 (83.2%)	1143 (82.8%)	
Body Mass Index (kg/m2)	28.6 (24.6, 33.6)	28.0 (24.5, 33.1)	28.0 (24.3, 32.6)	28.4 (24.6, 32.5)	0.464
Waist circumference (cm)	99 (87.87–109.6)	98.15 (88.47–109.4)	97.85 (87.75–109.9)	98.8 (89.3–110)	0.656
Triglycerides (mg/dl)	94 (65–139)	98 (65–144)	96 (66-142.3)	99 (66–149)	0.335
Fasting glucose (mg/dl)	100 (93–110)	101 (94-110.3)	101 (94–111)	102 (95–111)	0.316
High density cholesterol (mg/dl)	52 (42–63)	51 (43–63)	52 (43–62)	50 (41–61)	0.033
Systolic Blood pressure (mmHg)	120.7 (110.7-133.3)	120.7 (111.3–132)	120.7 (111.3-133.3)	120.7 (112-132.7)	0.671
Diastolic Blood pressure (mmHg)	70 (63.3–73.3)	70 (63.3–73.3)	71.3 (64–78)	71.3 (64.7–78)	0.066
Total energy intake (kcals)	1198 (892, 1589)	1737 (1390, 2117)	2087 (1644, 2582)	2598 (2053, 3298)	< 0.001
Vigorous activity, n(%)					
Yes	280 (20.2%)	256 (18.6%)	302 (21.9%)	386 (28.0%)	< 0.001
No	1104 (79.8%)	1124 (81.4%)	1078 (78.1%)	995 (72.0%)	
Moderate activity, n(%)					
Yes	500 (36.1%)	526 (38.1%)	548 (39.7%)	595 (43.1%)	0.002
No	884 (63.9%)	854 (61.9%)	832 (60.3%)	786 (56.9%)	

**Table 3:** Associations of choline intake with ASCVD, subgroup analysis stratified by heart failure, coronary heart disease, angina/angina pectoris, heart attack and stroke

	Quartile1 (*n* = 1384)	Quartile2(*n* = 1380)	Quartile3(*n* = 1380)	Quartile4(*n* = 1381)	
History of congestive heart failure, n(%)					
	54	32	30	35	0.02
	1330	1348	1350	1346	
History of coronary heart disease, n(%)					
	54	56	43	42	0.34
	1330	1324	1337	1339	
History of angina/angina pectoris, n(%)					
	30	33	30	18	0.18
	1354	1347	1350	1363	
History of heart attack, n(%)					
	57	54	45	45	0.506
	1327	1326	1335	1336	
History of stroke, n(%)					
	63	39	31	38	0.003
	1321	1341	1349	1343	

We also conducted a multivariable logistic regression analysis (Table 4). In the unadjusted model (model 1), individuals in the higher tertiles of choline intake exhibited a significantly reduced odds of stroke and ASCVD. However, no significant associations were observed between choline intake and heart disease (including congestive heart failure, coronary heart disease, angina/angina pectoris, and heart attack). After adjustment for age, gender, race, ethnicity, and physical activity in model 2, choline intake was found to be a protective effect for heart disease [Q4 vs. Q1, OR = 0.77, 95% CI = (0.53,1.01)], stroke [Q4 vs. Q1, OR = 0.69, 95% CI = (0.41,0.98)], and ASCVD [Q4 vs. Q1, OR = 0.74, 95% CI = (0.54,0.95)]. After further adjusting for drinking status, smoking status, fasting glucose, HDL-C, triglycerides, waist circumference, Body Mass Index and BP (Model 3), the multivariate logistic regression analysis revealed a significant association between choline intake and heart disease (*P* = 0.045), stroke (*P* = 0.032), and ASCVD (*P* = 0.022). Notably, we observed that the third quartile of choline intake was potentially associated with a lower odds of congestive heart failure and stroke. When further adjusted for all covariates (plus total energy intake, Model 4), choline in Q3 group was negatively correlated with the risk of heart disease (OR = 0.78), stroke (OR = 0.53), and ASCVD (OR = 0.68), while there were no statistically significant differences (*P* = 0.266 for heart disease; *P* = 0.182 for stroke; *P* = 0.159 for ASCVD). The interaction between sex and choline intake was not significant on the associations with ASCVD. However, sex showed significant interaction effects on the association between choline and coronary heart disease (p for interaction = 0.0258), choline and angina/angina pectoris (p for interaction = 0.035), suggesting that the impact of choline intake on the above two diseases may vary depending on an individual’s sex.

**Table 4:** Odd ratios of choline intake with atherosclerotic cardiovascular diseases (ASCVD)

OR (95% CI)	Heart disease	Stroke	ASCVD
Model 1			
Quartile1	1.00 (Reference)	1.00 (Reference)	1.00 (Reference)
Quartile2	0.90 (0.65, 1.15)	0.65 (0.40, 0.91)	0.82 (0.62, 1.03)
Quartile3	0.85 (0.62, 1.09)	0.52 (0.31, 0.74)	0.76 (0.57, 0.95)
Quartile4	0.81 (0.59, 1.04)	0.64 (0.39, 0.89)	0.78 (0.59, 0.98)
P trend	0.084	0.000311	0.00961
Model 2			
Quartile1	1.00 (Reference)	1.00 (Reference)	1.00 (Reference)
Quartile2	0.80 (0.57, 1.04)	0.65 (0.39, 0.91)	0.73 (0.54, 0.93)
Quartile3	0.72 (0.51, 0.93)	0.49 (0.28, 0.70)	0.63 (0.46, 0.81)
Quartile4	0.77 (0.53, 1.01)	0.69 (0.41, 0.98)	0.74 (0.54, 0.95)
P trend	0.00808	0.000145	0.00027
Model 3			
Quartile1	1.00 (Reference)	1.00 (Reference)	1.00 (Reference)
Quartile2	0.82 (0.60, 1.11)	0.75 (0.56, 0.99)	0.77 (0.56, 0.99)
Quartile3	0.72 (0.53, 0.99)	0.38 (0.43, 0.99)	0.64 (0.48, 0.85)
Quartile4	0.76 (0.55, 1.01)	0.49 (0.31, 0.77)	0.74 (0.56, 0.99)
P trend	0.04511	0.0322	0.0227
Model 4			
Quartile1	1.00 (Reference)	1.00 (Reference)	1.00 (Reference)
Quartile2	0.85 (0.62, 1.17)	0.67 (0.43, 1.03)	0.77 (0.58, 1.03)
Quartile3	0.78 (0.55, 1.10)	0.53 (0.32, 0.87)	0.68 (0.50, 0.92)
Quartile4	0.86 (0.58, 1.26)	0.81 (0.45, 1.33)	0.81 (0.58, 1.15)
P trend	0.26671	0.18194	0.15976
P for interaction ^a^	0.2145	0.3017	0.4777

Model 1 was adjusted for NHANES cycle (crude model)

Model 2 further controlled for age (continuous variable), gender, race and physical activity (binary variable)

In addition to model 2, model 3 further controlled for drinking status, smoking status (binary variable), fasting glucose, high density cholesterol, triglycerides, waist circumference, Body Mass Index, and blood pressure (continuous variable)

In addition to model 3, model 4 further controlled for drinking status, smoking status (binary variable), fasting glucose, high density cholesterol, triglycerides, waist circumference, blood pressure, Body Mass Index and Total energy intake (continuous variable)

a, P for interaction between sex and choline intake on ASCVD.

### Dose-response associations between choline and ASCVD

**Fig. 2:**
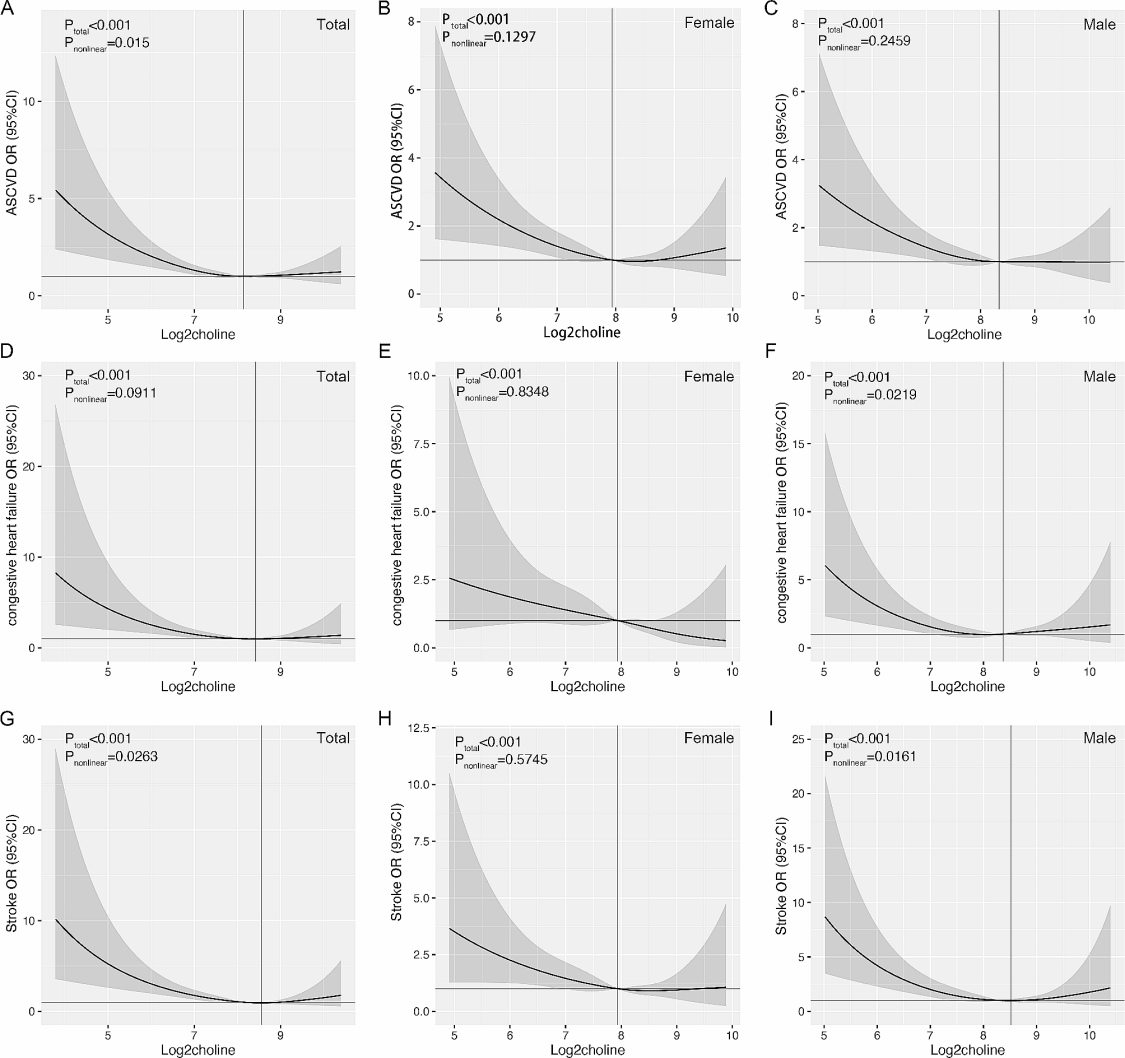
Restricted cubic spline analysis of dose-response relationships between choline and atherosclerotic cardiovascular diseases (ASCVD). (**A**) ASCVD for all participants; (**B**) ASCVD for female; (**C**) ASCVD for male; (**D**) congestive heart failure for all participants; (**E**) congestive heart failure for female; (**F**) congestive heart failure for male; (**G**) stroke for all participants; (**H**) stroke for female; (**I**) stroke for male. Results adjusted for age, race, and physical activity. The solid line represents the odds ratios, and the dotted line represents the 95% confidence interval

The data presented above suggest a possible non-linear relationship between choline and ASCVD. To investigate this further, we conducted a restricted cubic spline analysis to assess the dose-response relationship of choline. Figure 2A shows a mirrored J-shaped relationship between choline and ASCVD (P_nonlinear_=0.015). However, in the subgroup analysis, we did not observe significant dose-response relationship among women (P_nonlinear_=0.2459, Fig. 2B) or men (P_nonlinear_=0.005, Fig. 2C). In addition, no significant association between choline and congestive heart failure was observed among all participants (P_nonlinear_=0.0911, Fig. 2D) or women (P_nonlinear_=0.8348, Fig. 2E), and Fig. 2F showed a mirrored J-shaped relationship between choline and congestive heart failure in males (P_nonlinear_=0.0219). When looking into specific types of heart disease, including coronary heart disease, angina/angina pectoris and heart attacks, no significant associations were observed among all participants, female or male (Fig. S1). When referring to stroke, a mirrored J-shaped relationship was observed among all participants (P_nonlinear_= 0.0263, Fig. 2G) and males (P_nonlinear_= 0.0161, Fig. 2I), but no significant association among females (P_nonlinear_= 0.5745, Fig. 2H).

### Associations between choline and MetS and its components

We analyzed the relationship between choline intake and the risk of MetS. As shown in Table 2, no significant differences were observed in waist circumference, triglycerides, fasting glucose, SBP, and DBP across different tertiles of dietary choline intake. However, a decrease in HDL-C was observed in the upper quartile of choline concentration (*P* = 0.033). We then divided the participants into groups with and without MetS. Table 5 shows that age, race, educational level, drinking, and smoking were associated with an increased risk of MetS. Individuals with MetS had a significantly higher proportion of ASCVD (*P* < 0.01 for all). Notably, no statistically significant difference was observed in physical activity between the groups, and there was no significant association between choline intake and MetS (280.1 vs. 289.7 mg/d, *P* = 0.327).

**Table 5:** Associations between choline intake and metabolic syndrome (MetS)

	Total (*n* = 5525)	non-MetS (*n* = 4146)	MetS (*n* = 1379)	*P* value
Gender, n(%)				0.478
Female	2680 (48.5%)	2023 (48.8%)	657 (47.6%)	
Male	2845 (51.5%)	2123 (51.2%)	722 (52.4%)	
Age [mean (SD)]	48.0 ± 16.7	46.0 ± 16.8	54.0 ± 15.0	< 0.001
Race/ethnicity, n(%)				< 0.001
Mexican American	727 (13.2%)	511 (12.3%)	216 (15.7%)	
Non-Hispanic Black	1250 (22.6%)	957 (23.1%)	293 (21.2%)	
Non-Hispanic White	2204 (39.9%)	1624 (39.2%)	580 (42.1%)	
Other Hispanic	565 (10.2%)	408 (9.84%)	157 (11.4%)	
Other Race	779 (14.1%)	646 (15.6%)	133 (9.64%)	
Educational level, n(%)				< 0.001
Above high school	3268 (59.1%)	2552 (61.6%)	716 (51.9%)	
Below high school	347 (6.28%)	221 (5.33%)	126 (9.14%)	
High school	1910 (34.6%)	1373 (33.1%)	537 (38.9%)	
Marital status, n(%)				0.05
Living alone	2197 (39.8%)	1680 (40.5%)	517 (37.5%)	
Not living alone	3328 (60.2%)	2466 (59.5%)	862 (62.5%)	
Drinking status, n(%)				< 0.001
Never drinker	1080 (19.5%)	702 (17.0%)	378 (27.4%)	
Current drinker	4445 (80.5%)	3444 (83.0%)	1001 (72.6%)	
Fasting glucose (mg/dl)	110 (36.0)	102 (23.3)	135 (52.3)	< 0.001
High density cholesterol (mg/dl)	53.7 (16.0)	57.2 (15.5)	43.2 (12.6)	< 0.001
Smoking status, n (%)				< 0.001
Never smoker	2914 (52.7%)	2287 (55.0%)	627 (45.5%)	
Current smoker	2611 (47.3%)	1859 (45.0%)	752 (54.5%)	
Triglycerides (mg/dl)	120 (105)	95.4 (54.1)	194 (168)	< 0.001
Waist circumference (cm)	100 (16.8)	95.9 (15.3)	112 (15.0)	< 0.001
Body Mass Index (kg/m^2^)	28.2 (24.5, 33.0)	26.8 (23.6, 31.0)	32.3 (29.1, 37.4)	< 0.001
Systolic Blood pressure (mmHg)	123 (18.0)	120 (16.3)	134 (18.8)	< 0.001
Diastolic Blood pressure (mmHg)	70.6 (12.0)	69.4 (11.3)	74.2 (13.2)	< 0.001
Vigorous activity, n(%)				0.1
No	4301 (77.8%)	3205 (77.3%)	1096 (79.5%)	
Yes	1224 (22.2%)	941 (22.7%)	283 (20.5%)	
Moderate activity, n(%)				0.489
No	3356 (60.7%)	2507 (60.5%)	849 (61.6%)	
Yes	2169 (39.3%)	1639 (39.5%)	530 (38.4%)	
Total energy intake (kcals)	1861 (1357, 2465)	1874 (1377, 2480)	1808 (1300, 2426)	0.015
Choline intake (mg/d)	287.5 (188.9-416.8)	280.1 (185.3, 414.8)	289.75 (190.2, 417.8)	0.327
History of congestive heart failure, n(%)				< 0.001
No	5374 (97.3%)	4066 (98.1%)	1308 (94.9%)	
Yes	151 (2.73%)	80 (1.93%)	71 (5.15%)	
History of coronary heart disease, n(%)				< 0.001
No	5330 (96.5%)	4031 (97.2%)	1299 (94.2%)	
Yes	195 (3.53%)	115 (2.77%)	80 (5.80%)	
History of angina/angina pectoris, n(%)				< 0.001
No	5414 (98.0%)	4083 (98.5%)	1331 (96.5%)	
Yes	111 (2.01%)	63 (1.52%)	48 (3.48%)	
History of heart attack, n(%)				< 0.001
No	5324 (96.4%)	4027 (97.1%)	1297 (94.1%)	
Yes	201 (3.64%)	119 (2.87%)	82 (5.95%)	
History of stroke, n(%)				0.001
No	5354 (96.9%)	4036 (97.3%)	1318 (95.6%)	
Yes	171 (3.10%)	110 (2.65%)	61 (4.42%)	
ASCVD, n(%)				< 0.001
No	5015 (90.8%)	3848 (92.8%)	1167 (84.6%)	
Yes	510 (9.23%)	298 (7.19%)	212 (15.4%)	

We conducted further analysis to investigate the linear and non-linear relationship between choline and MetS. As anticipated, linear regression analysis revealed no significant linear relationship between choline and the components of MetS, including waist circumference, triglycerides, fasting glucose, and SBP (*P* > 0.05 for all). A slight positive correlation was observed between choline and DBP (β-coefficient = 1.002, *P* = 0.032, Table S3). Furthermore, the analysis included a smoothing estimate for the association between choline intake levels and MetS, adjusted for age, sex, and physical activity. The results show no significant association between choline intake levels and the occurrence of MetS (P_nonlinear_= 0.537, Figure S2).

## Discussion

In this study, we investigated the associations between choline intake and ASCVD in individuals from NHANES 2011–2018, while also exploring potential sex-dependent heterogeneities. After adjusting for multiple covariates, we observed that the third quartile of dietary choline intake may be associated with a lower odds of congestive heart failure and stroke. Our findings agree with the results from regression analysis, which revealed a mirrored J-shaped dose-response relationship between dietary choline and ASCVD. However, we did not observe any significant associations between choline intake and MetS or its components.

Although a weak relationship between choline and total heart disease was observed in this study, subgroup analysis revealed an inverse association between choline intake and congestive heart failure. The non-linear analysis suggested that certain amounts of choline may have protective effects in individuals. However, a slightly increased risk of congestive heart failure was observed when choline intake exceeded approximately 342 mg/d, indicating that excessive amounts of choline greater than what is needed may increase the risk of ASCVD. Contrary to our findings, the PREDIMED study cohort found that plasma choline was independently associated with increased risk of heart failure [6]. Supplementation with choline and its metabolite trimethylamine N-oxide (TMAO) promotes the development atherosclerosis and heart failure in animal studies [24, 25]. The SURDIAGENE cohort study found no statistically significant association between choline and heart failure in patients with type 2 diabetes [26]. The contradictory role of choline in cardiovascular disease is mainly attributed to the opinion that choline in the diet is metabolized to TMAO by the intestinal micro-organisms, and excessive choline intake leads to an increase in plasma TMAO levels [27]. Consuming a high-fat diet enriched with choline was found to negatively impact bioenergetics of mitochondria in the colonic epithelium. This led to an increase in the availability of oxygen and nitrate in the gut, which in turn intensified the breakdown of choline by Escherichia coli through respiration [28]. However, Wang et al. identified that carnitine, but not choline, increased plasma TMAO levels and discontinuation of dietary red meat could reduce TMAO levels within four weeks [29]. In addition, Zhu et al. suggested that consuming whole eggs raises plasma choline levels in overweight postmenopausal women, but does not impact TMAO levels. The above data refutes a causal relationship between dietary choline and elevated plasma TMAO. Choline bitartrate supplements significantly increase plasma choline and TMAO levels [30], indicating that the form and source of choline in the diet have varying effects on TMAO levels. The levels of choline intake in this study were calculated based on choline levels in different diets. However, there is a lack of information on TMAO leading to an inability to establish a direct relationship between choline intake and TMAO production. Therefore, large-scale clinical studies are still needed to directly establish a causal relationship between a high choline diet and high circulating TMAO levels.

Another finding of this study is that choline intake has a strong inverse relationship with stroke. Consistently, Zhong et al found plasma choline was inversely associated with cognitive impairment after stroke [31]. It has been reported that choline plays a role in neuro-development and the pathogenesis of multiple chronic diseases, as it is metabolically connected to both lipid and folate-dependent carbon metabolism [32]. As expected, choline deficiency causes an increase in plasma homocysteine [33] and dietary deficiency of choline in humans causes fatty liver and muscle damage [34]. There are significant variations in the amount of choline required for normal organ function among different individuals. While some individuals require more than the recommended adequate intake of 550 mg/d, others require less than 50 mg/d [35]. Deficiencies in folic acid and choline lead to larger damage, reduced neuro-degeneration and inflammation after ischemic stroke in middle-aged offspring [36]. Vice versa, citicoline and choline alphoscerate, which are phospholipids containing choline, have been suggested as adjuvants in the treatment of acute strokes [37]. In our study, subgroup analysis found high intake of choline seems to have fewer protective effects in females than that in males. Estrogen may be responsible for the increased synthesis of endogenous phosphatidylcholine, which could account for the lower incidence of organ dysfunction symptoms in premenopausal women compared to men and postmenopausal women [38]. However, we need to interpret the data cautiously because there is no significant interaction between sex and choline intake in the logistic regression analysis for ASCVD. The presence of sex-specific associations and the physiological mechanisms behind them warrant further investigation. In sum, our study, for the first time using NHANES data, reveals clinical evidence that adequate choline intake (approximate 244 mg/d for females and 367 mg/d for males) showed substantive benefits in both sexes. What’s more, our data found that unlike ASCVD, excessive choline intakes didn’ t significantly increase the risk of stroke. The reason may be that the brain could prevent excessive choline intake through rapid metabolism and adaptive, diet-induced changes in choline net absorption and release [39].

It is estimated that dietary factors are associated with a substantial proportion of deaths from heart disease, and stroke [40]. Metabolic syndrome (MetS) is a reliable predictor of all-cause mortality of ASCVD. A series of longitudinal data have demonstrated that MetS is a risk factor for ASCVD [41, 42]. Despite the availability of the NHANES database, no research has yet examined the correlation between choline and MetS. The present study aimed to reveal the association between dietary choline and MetS, and its possible impact on cardiometabolic disease in the U.S. Surprisingly, the data revealed no significant linear or non-linear association between choline intake and MetS. Only a slight positive correlation was found between choline intake and DBP. By contrast, a recent study reported that a higher dietary intake of choline is associated with lower levels of blood pressure and LDL concentration among obese individuals [17]. Taesuwan et al. found no correlation between choline consumption and either systolic or diastolic blood pressure [18, 19]. That study included only a small number of obese people which is far fewer than in our study. We included 4206 (76.1%) with abdominal obesity (2165 males had a waist circumference > 90 cm, and 2041 females > 85 cm). Although our large study observed an absence of a relationship between choline and BP, intervention studies are still needed in order to determine whether a high choline-diet is involved in the progression of hypertension.

This study has some noteworthy limitations. The first one is that the levels of choline intake in the datasets were obtained through a questionnaire that assesses the types and quantities of foods and beverages (including all types of water) consumed during the day, and the levels of choline from these foods and beverages is calculated. The accuracy of this, however, remains unclear as there are important limitations associated with the absorption and metabolism of choline [43]. Perhaps it is precisely because of the first limitation that our sensitivity analysis found that after adjusting for total energy intake, the protective effect of choline on ASCVD showed no statistical significance. This is not surprising because choline intake is also part of overall energy intake; they both have several similar components, such as eggs, red meat, fruits, vegetables, and whole grains. Another limitation is that NHANES datasets doesn’t contain data on plasma TMAO levels and we were not able to analyze direct causality between choline intake and TMAO in U.S. adults. Further, this is a cross-sectional study and we ought to exercise caution before ascertaining that our results exhibit causal relations. Specifically, the observed relationship between choline consumption and ASCVD could potentially be influenced by residual confounding. The longitudinal studies would be suggested to assess a causal relationship between choline intake and ASCVD. Last but not least, cardioprotective effects of choline need to be confirmed by intervention studies and possible harmful effects of excessive choline on cardiometabolic health need careful evaluation.

## Conclusion

In the current analysis of NHANES participants from 2011 to 2018, choline was inversely and non-linearly associated with ASCVD in American adults. This relationship was more pronounced in females. The findings underscore the significance of developing individualized dietary recommendations in reducing the risk of ASCVD.

### Electronic supplementary material

Below is the link to the electronic supplementary material.

Supplementary Material 1

## Data Availability

The datasets used for these analyses are publicly available (https://wwwn.cdc.gov/nchs/nhanes).

## Abbreviations

ASCVD: Atherosclerotic cardiovascular disease
NHANES: National Health and Nutrition Examination Survey
MetS: Metabolic syndrome
