# Supplementary material for: An inverse association of dietary choline with atherosclerotic cardiovascular disease among US adults: a cross-sectional NHANES analysis
Source: BMC Public Health. 2024 May 31;24:1460. doi: 10.1186/s12889-024-18837-8 (PMC11141004; doi:10.1186/s12889-024-18837-8)
Supplement: Supplementary file 1 — Supplementary Material 1 [file 12889_2024_18837_MOESM1_ESM.docx]

Supplementary Material

**Dietary choline is inversely associated with atherosclerotic cardiovascular disease: A cross-sectional study of the National Health and Nutrition Examination Survey (NHANES) 2011 to 2018**

Supplementary Table 1. The choline content of common foods.

Supplementary Table 2. Disease diagnosis from NHANES database.

Supplementary Table 3. Subgroup analysis of the association between choline and congestive heart failure, coronary heart disease, angina/angina pectoris, heart attack.

Supplementary Figure 1. Graphics of smooth curve fittings of choline intakes and atherosclerotic cardiovascular diseases (ASCVD).

Supplementary Table 4. Linear regression analysis for the associations between choline intake and the components of metabolic syndrome (MetS).

Supplementary Figure 2. Graphics of smooth curve fittings of choline intakes and metabolic syndrome (MetS).

**Supplementary Table 1. the choline content of common foods.**

| category | mg total choline/100 g food |
| --- | --- |
| Whole eggs | 250 |
| Meats & fish | 80 |
| Whole grains | 67 |
| Breakfast cereal | 50 |
| Vegetables and fruits | 30 |
| Milk | 20 |
| Fats and oils | 5 |

More detailes could be found on the United States Department of Agriculture (USDA) food content databases (https://data.nal.usda.gov/dataset/usda-database-choline-content-common-foods-release-2-2008).

# Supplementary Table 2. Disease diagnosis from NHANES database.

| **Disease** | **Section** | **English Text** | **Value Description for Diagnosis Yes** |
| --- | --- | --- | --- |
| angina/angina pectoris | Questionnaire Data | Has a doctor or other health professional ever told {you/SP} that {you/s/he} . . .had angina (an-gi-na), also called angina pectoris? | Yes |
| heart attack | Questionnaire Data | Has a doctor or other health professional ever told {you/SP} that {you/s/he} . . .had a heart attack (also called myocardial infarction (my-o-car-dee-al in-fark-shun))? | Yes |
|  | Questionnaire Data | Has a doctor or other health professional ever told {you/SP} that {you/he/she} had …? | a heart attack? |
| stroke | Questionnaire Data | Has a doctor or other health professional ever told {you/SP} that {you/s/he} . . .had a stroke? | Yes |
|  | Questionnaire Data | Has a doctor or other health professional ever told {you/SP}that {you/he/she} had . . .? | a stroke? |
| coronary heart disease | Questionnaire Data | Has a doctor or other health professional ever told {you/SP} that {you/s/he} . . .had coronary (kor-o-nare-ee) heart disease? | Yes |
| hypertension | Questionnaire Data | {Were you/Was SP} told on 2 or more different visits that {you/s/he} had hypertension, also called high blood pressure? | Yes |
|  | Questionnaire Data | {Have you/Has SP} ever been told by a doctor or other health professional that {you/s/he} had hypertension, also called high blood pressure? | Yes |
|  | Examination Data | Average SBP, average DBP (at least 3 times) | Average SBP≥140 mmHg, average DBP≥90 mmHg |
|  | Questionnaire Data | Use of antihypertensive medication, including angiotensin converting enzyme inhibitors, angiotensin receptor blockers, diuretics, calcium channel blockers, beta blockers, alpha blockers, centrally acting agents, direct vasodilators, aldosterone receptor antagonists, renin inhibitors, other antihypertensive agents. |  |

**Supplementary Table 3. Subgroup analysis of the association between choline and congestive heart failure, coronary heart disease, angina/angina pectoris and heart attack.**

| OR (95% CI) | congestive heart failure | coronary heart disease | angina/angina pectoris | heart attack |
| --- | --- | --- | --- | --- |
| Model 1 |  |  |  |  |
| Quartile1 | Reference | Reference | Reference | Reference |
| Quartile2 | 0.58 (0.37, 0.91) | 1.04 (0.71, 1.53) | 1.10 (0.67, 1.83) | 0.95 (0.65, 1.39) |
| Quartile3 | 0.55 (0.34, 0.85) | 0.79 (0.52, 1.19) | 1.00 (0.60, 1.68) | 0.78 (0.52, 1.17) |
| Quartile4 | 0.64 (0.41, 0.98) | 0.77 (0.51, 1.16) | 0.60 (0.24, 1.06) | 0.78 (0.52, 1.16) |
| P trend | 0.0464 | 0.0364 | 0.0855 | 0.125 |
| Model 2 |  |  |  |  |
| Quartile1 | Reference | Reference | Reference | Reference |
| Quartile2 | 0.53 (0.33, 0.83) | 0.91 (0.60, 1.36) | 1.02 (0.61, 1.70) | 0.85 (0.57, 1.27) |
| Quartile3 | 0.47 (0.29, 0.75) | 0.63 (0.41, 0.97) | 0.87 (0.51, 1.47) | 0.66 (0.43, 1.00) |
| Quartile4 | 0.63 (0.39, 0.98) | 0.67 (0.43, 1.04) | 0.57 (0.30, 1.03) | 0.72 (0.47, 1.11) |
| P trend | 0.0637 | 0.0143 | 0.0824 | 0.1005 |
| Model 3 |  |  |  |  |
| Quartile1 | Reference | Reference | Reference | Reference |
| Quartile2 | 0.55 (0.34, 0.87) | 0.98 (0.65, 1.48) | 1.09 (0.65, 1.84) | 0.93 (0.62, 1.39) |
| Quartile3 | 0.47 (0.29, 0.76) | 0.66 (0.42, 1.03) | 0.91 (0.53, 1.55) | 0.71 (0.46, 1.08) |
| Quartile4 | 0.65 (0.40, 1.04) | 0.69 (0.44, 1.08） | 0.60 (0.32, 1.10) | 0.78 (0.50, 1.20) |
| P trend | 0.03881 | 0.03829 | 0.0926 | 0.14132 |
| Model 4 |  |  |  |  |
| Quartile1 | Reference | Reference | Reference | Reference |
| Quartile2 | 0.62 (0.38, 1.00) | 1.00 (0.65, 1.53) | 1.13 (0.66, 1.93) | 0.98 (0.64, 1.49) |
| Quartile3 | 0.59 (0.34, 0.99) | 0.68 (0.42, 1.10) | 0.96 (0.53, 1.73) | 0.77 (0.48, 1.23) |
| Quartile4 | 0.93 (0.52, 1.62) | 0.72 (0.44, 1.23） | 0.65 (0.31, 1.32) | 0.91 (0.54, 1.51) |
| P trend | 0.5991 | 0.11420 | 0.2479 | 0.4672 |
| P for interaction^a^ | 0.0573 | 0.0258 | 0.03514 | 0.33608 |

Model 1 was adjusted for NHANES cycle (crude model).

Model 2 further controlled for age (continuous variable), gender, race and physical activity (binary variable).

In addition to model 2, model 3 further controlled for drinking status, smoking status (binary variable), fasting glucose, high density cholesterol, triglycerides, waist circumference, Body Mass Index, and blood pressure (continuous variable).

In addition to model 3, model 4 further controlled for drinking status, smoking status (binary variable), fasting glucose, high density cholesterol, triglycerides, waist circumference, blood pressure, Body Mass Index and Total energy intake (continuous variable).

a P for interaction between sex and choline intake on ASCVD.

#

# Supplementary Figure 1. Graphics of smooth curve fittings of choline intakes and atherosclerotic cardiovascular diseases.


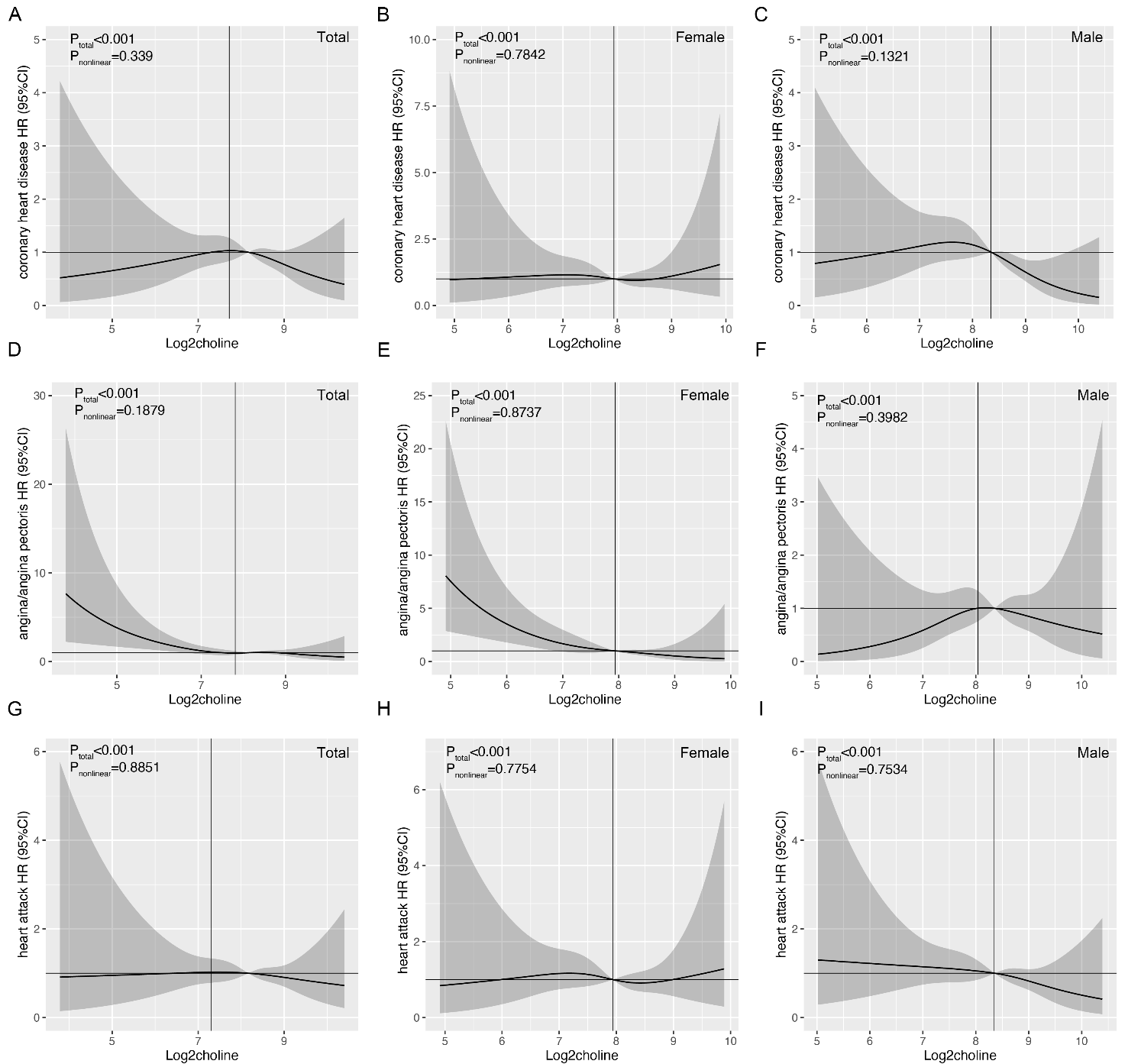


# Figure S1. Restricted cubic spline analysis of dose-response relationships between choline and atherosclerotic cardiovascular diseases (ASCVD). (A) coronary heart disease for all participates; (B) coronary heart disease for female; (C) coronary heart disease for male; (D) angina/angina pectoris for all participates; (E) angina/angina pectoris for female; (F) angina/angina pectoris for male; (G) heart attack for all participates; (H) heart attack for female; (I) heart attack for male. Results adjusted for age, race, and physical activity. The solid line represents the odds ratios, and the dotted line represents the 95% confidence interval.

**Supplementary Table 4.** Linear regression analysis for the associations between choline intake and the components of metabolic syndrome (MetS).

| Outcomes | β-coefficient | 95% CI | P value |
| --- | --- | --- | --- |
| Waist circumference (cm) | 1.00052 | (-0.000897, 0.001915) | 0.478 |
| Systolic Blood pressure (mmhg) | 0.99983 | (-0.001473, 0.001151) | 0.810 |
| Diastolic Blood pressure (mmhg) | 1.00216 | (0.0001840, 0.004131) | 0.032 |
| Fasting glucose (mg/dl) | 1.00012 | (-0.000527, 0.000785) | 0.700 |
| High density cholesterol (mg/dl) | 0.99890 | (-0.002568, 0.000383) | 0.147 |
| Triglycerides (mg/dl) | 0.00015 | (-0.000072, 0.000376) | 0.185 |

# Supplementary Figure 2. Graphics of smooth curve fittings of choline intakes and metabolic syndrome (MetS).


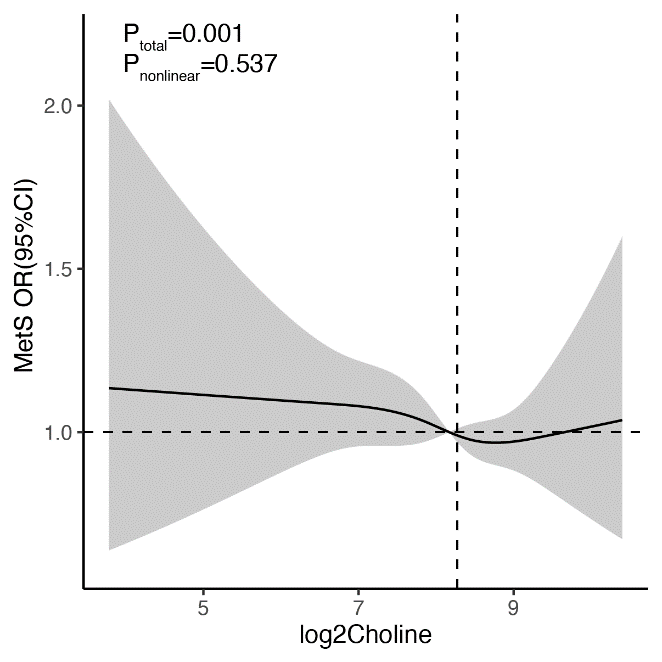


**Figure S2. Restricted cubic spline analysis of dose-response relationships between choline and metabolic syndrome.**
